# A single center prospective study: Influences of different hip flexion angles on the measurement of lumbar spine bone mineral density by dual energy X-ray absorptiometry

**DOI:** 10.1515/med-2023-0778

**Published:** 2023-09-12

**Authors:** Lisheng Yan, Donglu Cai, Huafeng Zhuang, Yongjun Lin

**Affiliations:** Department of Radiology, The Second Affiliated Hospital of Fujian Medical University, QuanZhou, 362000, China; Department of Orthopedics, The Second Affiliated Hospital of Fujian Medical University, No. 950 Donghai Street, Fengze District, Quanzhou 362000, Fujian, China; Department of Orthopedics, The Second Affiliated Hospital of Fujian Medical University, Quanzhou 362000, Fujian, China

**Keywords:** dual energy X-ray absorptiometry, bone mineral density, lumbar spine, hip flexion angle, osteoporosis

## Abstract

To investigate whether there is an influence on the results of lumbar spine bone mineral density (BMD) measured by dual-energy X-ray absorptiometry (DXA) under three different hip flexion angles (90°, 45°, 0° of hip flexion). We collected a total of 60 outpatients, including 44 females (56.4 ± 5.7 years) and 16 males (50.2 ± 13.7 years). The DXA results of the lumbar spine were scanned and analyzed in three different positions with hip flexion of 90°, 45°, and 0°. We found that there was no significant difference in the area of interest, bone mineral content, BMD, and vertebral body height of the lumbar vertebral body measured by DXA in three hip flexion positions of 90°, 45°, and 0°; Pearson’s correlation analysis showed that lumbar BMD in hip flexion 90° was correlated with it in hip flexion 45° (*r* = 0.998, *P*＜0.01) and in hip flexion 0° (*r* = 0.996, *P*＜0.01) respectively. There was no statistically significant difference in the diagnosis of BMD between 90° and 45° hip flexion (*P* = 0.903), which was the same as 90° and 0° hip flexion (*P* = 0.822). Therefore, we conclude that different hip flexion angles can be used in lumbar BMD detection by DXA, which is beneficial to patients who have difficulty in hip flexion, especially for elderly patients with osteoporosis.

## 1 Introduction

Dual-energy X-ray absorptiometry (DXA) is a commonly used bone mineral density (BMD) measurement method in clinical settings. It is essential for the diagnosis of osteoporosis, the risk assessment of osteoporotic fractures, and therapeutic monitoring [1]. Therefore, achieving a maximum DXA accuracy is primordial since it can significantly impact crucial outcome assessments such as efficacy monitoring and treatment effects. Yet, clinicians often encounter patients unable to meet the standard 90° hip flexion posture required by lumbar BMD scan protocols due to several reasons, such as the initial fracture or the related pain. Such situations have always been the subject of concern among imaging technicians since it is unclear whether a non-standard posture can affect the measurement results, subsequently leading to misdiagnosis or missed diagnosis. This work aims to explore whether variations in hip flexion angle have a significant impact on lumbar BMD measurements using DXA.

## 2 Materials and methods

### 2.1 Research participants

Data from 60 subjects who underwent DXA scans in our hospital’s outpatient department from November 1, 2021 to November 31, 2021 were collected and analyzed. All participants were between 42 and 75 years old, with an average age of 54.7 ± 7.8 years and a body mass index of 22.4 ± 2.3 kg/m^2^. Forty-four of the included patients were females, and 16 were males, with average ages of 56.4 ± 5.7 and 50.2 ± 13.7 years and body mass indexes of 22.0 ± 1.8 and 23.3 ± 3.5 kg/m^2^, respectively. This study was preapproved by The Ethics Committee of The Second Affiliated Hospital of Fujian Medical University. All participants and their families approved the procedures.

### 2.2 Inclusion criteria

All patients received in our hospital and requiring lumbar spine BMD measurements using DXA.

### 2.3 Exclusion criteria

(1) Patients with irremovable biomaterials such as bone cement or metal implants within their lumbar spines; (2) subjects suffering from lumbar spine disorders including severe scoliosis and lumbar osteophytes, lumbar spondylolisthesis, and a history of lumbar fracture; (3) a history of neoplastic growth; (4) recent use of drugs or medical diagnostic tests that affect bone metabolisms; and (5) an unclear scan due to various reasons.

### 2.4 Instruments and methodology

The DXA Discovery A model was purchased from Hologic, Inc. (Massachusetts, USA). Daily quality controls and repeated prechecks were meticulously performed before use to ensure instrument stability. The CV value of our machine was 0.244%. All scanning and analysis procedures were performed by the same International Society for Clinical Densitometry (ISCD)-trained technician, and all scans carried out on a patient were completed within 24 h. Scans were obtained with the patient posed at three different hip flexion angles, including 90°, 45°, and 0°. The standard 90° hip flexion images were acquired with the patient in the supine position at the center of the scanner bed, his hip on the positioning device and bent at a 90° angle, and his feet resting on the apparatus. The scan starting point was set at the level of the fifth lumbar vertebra before imaging and data collection. The 45° hip flexion images were obtained after those of the standard 90° positioning. The patient was maintained in the same posture as previously mentioned, with the minor difference of having his knees bent. The scan starting point was still at the level of the fifth lumbar vertebra. Finally, the 0° hip flexion data were collected with the patient lying in the same posture and having his legs resting straightly on the scanner bed. The scan starting point was also set at the level of the fifth lumbar vertebra. The required standard for the viability and analysis of various posture images was as follows: an excellent lumbar spine scan obtained following the protocol, the projection of the spinous process within the center of the vertebral body, clear visibility of the bilateral iliac crests, an upper limit encompassing the middle section of the T12 and a lower limit including the L5, a clearly displayed image. The following standard was used for the analysis of the three angle variations data: (1) the region of interest (ROI) was defined as the area with the upper limit located just above the L1 endplate and the lower limit situated just below the L4 endplate, (2) L1–L4 vertebral contours were considered as the lumbar spine edges during the scans, (3) the intervertebral line was placed in the center of the space between the two vertebral bodies. Finally, the three postures data comprised of several lumbar BMD parameters such as bone area, vertebral bone mineral content (BMC), vertebral BMD, and total vertebral body height were collected, compared, and analyzed.

### 2.5 Statistical analysis

All data were analyzed using SPSS 25.0 statistical software (IBM Software, Chicago, IL, USA). Measurement data were expressed as mean ± standard deviation. The paired *t*-test was used to compare the measurement data between different postures. Meanwhile, the comparison of count data between different body positions was performed using the contingency table chi-square analysis. Correlations between various hip flexion angles were assessed using Pearson’s correlation method. *P* < 0.05 was considered statistically significant.

**Figure 1:**
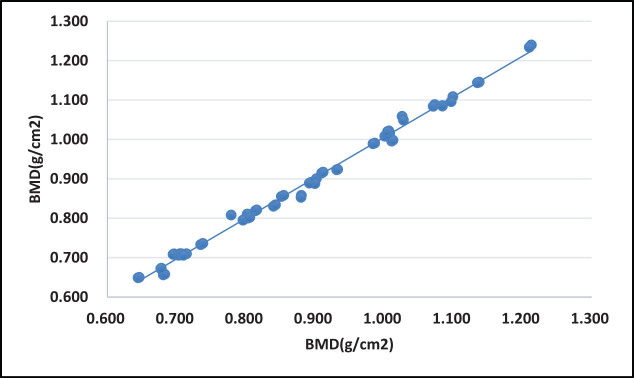
Correlation of vertebral BMD between hip flexion 90° and 45°.

**Figure 2:**
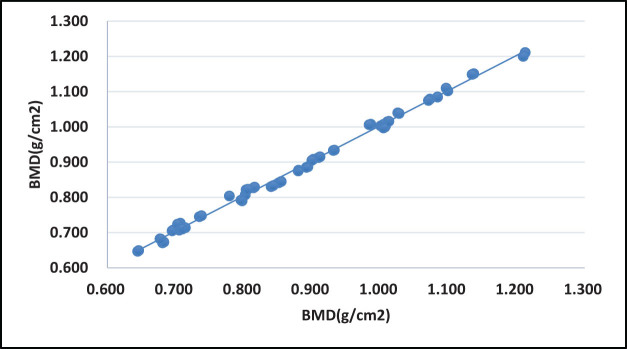
Correlation of vertebral BMD between hip flexion 90° and 0°.

**Table 1:** Comparison of DXA measurements in 90° and 45° hip flexion

	90° hip flexion	45° hip flexion	*t*	*P*
Area	59.09 ± 7.14	59.01 ± 7.11	0.901	0.375
BMC	52.58 ± 11.84	52.65 ± 12.13	−0.505	0.617
BMD	0.888 ± 0.156	0.890 ± 0.162	−1.123	0.271
Total vertebral body height	131.4 ± 6.1	131.2 ± 6.1	1.542	0.134

**Table 2:** Comparison of DXA measurements in 90° and 0° hip flexion

	90° hip flexion	0° hip flexion	*t*	*P*
Area	59.09 ± 7.14	58.91 ± 7.2	1.610	0.118
BMC	52.58 ± 11.84	52.4 ± 11.75	−1.437	0.162
BMD	0.888 ± 0.156	0.890 ± 0.156	−1.938	0.062
Total vertebral body height	131.4 ± 6.1	131.1 ± 6.2	1.882	0.070

**Table 3:** Comparison of BMD diagnosis in different hip flexion positions

Angle of hip flexion	Diagnosis of BMD			*χ* ^2^	*P*
	Normal (*n*)	Osteopenia (*n*)	Osteoporosis (*n*)		
90°	30	14	16		
45°	28	16	16	0.2023	0.903*
0°	28	17	15	0.3915	0.822^#^

P*: Comparison between hip flexion 90° and 45°; *P* ^#^: comparison between hip flexion 90° and 0°.

## 3 Results

1. No statistically significant differences in the ROI, vertebral BMC, vertebral BMD, and total vertebral body heights were observed between the 90° and the 45° angles (*P* > 0.05) as well as the 90° and the 0° hip flexion postures (*P* > 0.05) (Tables 1 and 2).
2. The BMD correlation analyses yielded coefficients of 0.998 and 0.996 between the 90° and the 45° angles, as well as the 90° and the 45° hip flexion postures, respectively. Additionally, the results showed significant correlations between the 90° and the remaining two angle variations (*P* < 0.05) (Figures 1 and 2).
3. Furthermore, the results showed that there were no significant differences in the diagnostic outcomes of BMD results between both the 90° and the 45° angles (*P* = 0.903) as well as the 90° and the 0° hip flexion postures (*P* = 0.822) (Table 3).

## 4 Discussion

The aging population in China is currently becoming an immediate and serious public health concern. The latest census data have shown that 260 million individuals are above 60 years, accounting for 18.70% of the overall population. Additionally, of the 260 million, 13.50% or 190 million individuals are above 65 years. Yet, osteoporosis is a major affliction impacting the elderly, with serious consequences such as cancellous fractures. Indeed, previous predictive studies have suggested that there will be around 5.99 million cases of osteoporosis by 2050 [2]. DXA is currently one of the primary means for the diagnosis of osteoporosis, with an 1A grading evidence quality and recommendation strength [3]. Therefore, its importance is further stressed by the observed trends in the population age curve, osteoporosis prevalence, and osteoporotic fracture incidences. Interestingly, research [4] has shown that variations and errors in DXA measurements are often human-made (operator or patient-derived) rather than the products of the machine itself. All 60 patients in our work were scanned by the same ISCD-certified technician, significantly reducing operator-related factors and increasing the test results’ accuracy and reliability, thus, leaving the patient’s posture as one of the major unaccounted influencing factors. The vast majority of daily encountered BMD test population are elderly patients suffering from several severe conditions such as arthritis, fractures, pain, and other mobility affecting afflictions, rendering them uncooperative or unable to comply with the 90° hip flexion posture required by the lumbar BMD examination. Therefore, it is imperative that answers to questions such as whether non-standard postures affect the accuracy of BMD measurements become the main subjects of focus.

Lekamwasam [5], in his analysis and comparison of lumbar BMD measurements from 56 postmenopausal women in the supine and the 90° hip flexion positions, discovered that there were no significant differences in the measured variables and obtained *t* values between the two positions. Coincidentally, Ikegami [6] also collected lumbar BMD data from 878 women and 161 men. The measurements were obtained with the patients in the supine and 90° hip flexion positions. The results confirmed that there were no significant differences in lumbar BMD between the two positions at various ages. Additionally, the findings also showed that the variation in hip position (supine and 90° flexion) did not impact the diagnostic rate of osteoporosis. In light of some pathological conditions that can lead to the patient’s inability to comply with the supine or the 90° hip flexion required, we proceeded to add a 45° flexion angle to expand BMD patients’ options. Our study showed that the variations in hip flexion angles (90°, 45°, and 0°) did not significantly affect parameters such as lumbar area of interest, BMC, BMD, and vertebral body height. Additionally, the lumbar BMD correlation results between the two postures (90° and 45°, 90° and 0°) were highly positive, with no significant differences in osteoporosis diagnostic rate between the various positions. The reason explaining our findings might be that even though the hip flexion angle varied significantly (from 90° to 0°), there is only a minimal change in lumbar curvature observed on the DXA two-dimensional image projection at different positions [7]. Furthermore, the changes in lumbar curvature are much more negligible, especially in elderly patients, due to loss of lumbar mobility and preexisting conditions such as degeneration. This is further supported by our vertebral body height comparison findings.

The current results suggested that there is no significant correlation between the lumbar BMD measurement results and the variations in hip flexion angles. Therefore, aiming to improve patients’ satisfaction and the test’s availability, we put forward the idea of safely adopting alternative hip flexion angles in clinical settings when the patient is unable to comply with the standard 90° posture since such changes have little effect on lumbar BMD measurement results. Additionally, it was found, during our study that the 45° and 0° hip flexion angles are not only more practical but also significantly improve work efficiency since they eliminate the need for a hip positioning device as well as shorten the scanning duration. However, it should also be pointed out that the 90° angle has the advantage of better image quality and is more suitable for image analysis since it reduces the possibility of a lumbar vertebral body and intervertebral space overlap. In summary, we believe that technicians should intuitively decide which posture to adopt on a case-to-case basis according to their patients’ specific situations.

## 5 Limitations

Our research had several limitations, including relatively small sample size and the use of a single DXA (Hologic, Inc., Massachusetts, USA), which might not be reflective of other brands.

## 6 Conclusions

Adaptive variations in hip flexion angles have minimal impacts on the outcome and are beneficial to DXA-measured lumbar BMD patients with hip flexion difficulties, especially the middle-aged and elderly subjects.
